# Endometrial gland remodeling and preserved luminal ultrastructure in postpartum cows infused with ozonated sunflower oil

**DOI:** 10.1590/1984-3143-AR2026-0001

**Published:** 2026-06-15

**Authors:** Jair Camargo Ferreira, Matheus dos Santos Oliveira, Cristiane Marisa Piacitelli Prado Ferreira, Maysa Barbosa de Almeida, Heloisa Andrade Rodrigues, Yatta Linhares Boakari, Marcela Aldrovani Rodrigues

**Affiliations:** 1 Programa de Pós-graduação em Ciência Animal, Universidade de Franca, Franca, SP, Brasil; 2 Department of Large Animal Clinical Sciences, Texas A&M School of Veterinary Medicine & Biomedical Sciences, College Station, TX, USA

**Keywords:** bovine reproduction, endometrial histomorphometry, non‑antibiotic therapy, uterine disease, scanning electron microscopy, puerperium

## Abstract

Postpartum uterine disease is a major cause of impaired bovine reproduction and is usually managed with intrauterine antibiotics. Alternative non‑antibiotic therapy is needed to support uterine health without contributing to antimicrobial resistance. The goal of this study was to characterize histomorphometric and ultrastructural changes of the bovine endometrium after intrauterine infusion of ozonated sunflower oil during early puerperium, and to assess its in vitro antimicrobial activity against uterine bacteria. Eighteen primiparous cows received a single intrauterine treatment with ozonated or non‑ozonated oil (O_3_ and non‑O_3_ groups; n=9/group) on day 10 postpartum. Endometrial biopsies were collected before and 15 days post-treatment (D0 and D15) for light microscopy and scanning electron microscopy. Uterine cytology was used to evaluate subclinical endometritis. Minimum inhibitory and bactericidal concentrations (MIC and MBC) of the treatments were determined against uterine isolates (*Escherichia coli*, *Staphylococcus* spp., *Streptococcus* spp. and *Arcanobacterium pyogenes*). At D15, the number of endometrial glands was higher in the O_3_ group than in the non‑O_3_ group (32.6±2.5 vs. 11.0±1.0; *P*<0.01), whereas glandular diameter, luminal area and cell number per gland were lower in O_3_ cows (*P*<0.01), indicating enhanced gland proliferation with reduced glandular dilatation. No degenerative histopathological changes, such as periglandular fibrosis or endometrial atrophy, were detected in either group, and scanning electron microscopy showed preserved apical ultrastructure without adverse effects of ozonated oil. Ozonated oil inactivated all uterine bacteria (MIC and MBC ≤0.18 µg mL^‑1^), while non‑ozonated oil showed no antimicrobial activity. Both groups showed a marked reduction in neutrophil percentage by D15, but histological evidence of persistent inflammation was more pronounced in non‑O_3_ cows. These findings indicate that intrauterine ozonated sunflower oil is a promising non‑antibiotic therapy to support postpartum uterine involution and endometrial recovery in dairy cows and may contribute to improved reproductive performance.

## Introduction

Endometritis is among the most prevalent disorders observed during the postpartum period in cattle (Sheldon and Owens, 2017). In addition to localized inflammation, the uterine lumen of nearly all cows becomes contaminated with bacteria during the first days after calving, although only a subset will develop persistent infection and clinical disease (Sheldon et al., 2008). Clinical and subclinical endometritis are typically diagnosed in approximately 20% and 30% of cows, respectively, beyond three to five weeks postpartum, depending on herd management, diagnostic criteria and time of examination (Cheong et al., 2011; Gilbert et al., 2005). Chronic or persistent endometritis markedly impairs subsequent reproductive performance, being associated with delayed uterine involution, prolonged luteal phases, reduced conception rates and extended calving intervals (de Boer et al., 2015; Ghanem et al., 2015).

Optimal management of uterine disease after parturition aims to eliminate pathogenic bacteria while preserving uterine immune function and minimizing economic losses related to milk withdrawal and impaired fertility. At present, treatment of postpartum uterine disorders in dairy cows still relies largely on systemic or intrauterine antibiotic therapy, particularly cephalosporins and penicillins (Andretta et al., 2022). However, antimicrobial use in this context is associated with drug residues in milk and meat and contributes to the emergence and spread of multidrug‑resistant pathogens in food‑producing animals (Várhidi et al., 2024). In light of these concerns, non‑antibiotic strategies, including ozone‑based formulations, have been proposed as adjunct approaches for managing inflammatory and infectious uterine diseases in cattle (Lefebvre, 2022).

Ozone (O_3_) therapy stimulates the synthesis of cellular growth factors without inducing adverse effects (Bocci, 2012). In mares, uterine O_3_ insufflation reduces local inflammation (Ávila et al., 2022) and triggers endometrial angiogenesis (Ferreira et al., 2021a). Nevertheless, widespread use of O_3_ in livestock herds is limited by the intrinsic instability of its gaseous form (Bocci, 1999) which requires on‑site generation, precise handling and specialized equipment for safe application.

In other hand, vegetable oils enriched with O_3_ are potent oxidizing agents with broad-spectrum germicidal action (Bocci, 2012), anti‑inflammatory effects (Xiao et al., 2021) and stable physicochemical properties for extended periods (Boland-Nazar et al., 2016). Given the immunomodulatory and antimicrobial properties attributed to ozonated sunflower oil, its intrauterine infusion during the puerperium may support uterine involution in dairy cows. However, information on local microscopic changes in the bovine endometrium in response to ozonated oil therapy during the postpartum period remains scarce. Therefore, the primary aim of the present study was to characterize the endometrial response to intrauterine infusion of ozonated sunflower oil in puerperal cows. The specific objectives were (a) to describe the morphometric and morphological changes of the endometrium exposed to ozonated oil during the early postpartum period and (b) to determine the in vitro antimicrobial activity of ozonated sunflower oil against uterine bacteria isolated from postpartum cows.

## Methods

### Animals

Initially, 30 postpartum mixed‑breed cows, 30–36 months of age and weighing 300–350 kg, were housed in an open shelter and outdoor paddock at the Research and Development Center located in Patrocínio Paulista, São Paulo, Brazil. Only primiparous cows with eutocic calving, no retained placenta, and no abnormal vaginal discharge were selected for the study. Management of the cows followed the guidelines provided in the Science Vet Guide for the Care and Use of Agricultural Animals in Research (FASS, 2020). All procedures were approved by the institutional Ethics Committee (protocol number #01/2024‑CEUA).

### Treatments and experimental groups

A total of 18 postpartum primiparous cows were selected and allocated to two treatment groups (O_3_ and non‑O_3_; n=9 cows/group). In the O_3_ group, a single intrauterine infusion of 50 mL of ozonated sunflower oil with a high peroxide index (>600 mmol‑meq kg^‑1^) was performed using a sterile insemination pipette. In the non‑O_3_ group, a similar procedure was performed using 50 mL of non‑ozonated sunflower oil (peroxide index <20 mmol‑meq kg^‑1^). The peroxide index of the oils was determined using a modified iodometric titration method, adapted from procedures established for highly ozonized oils (Cirlini et al., 2012) and ISO 3960:2017 (ISO, 2017).

Treatments were administered 10 after calving (10.8±0.5 days) by the same operator. Before treatment, the tail was wrapped, and the perineal area was washed, rinsed with clean water, and dried with disposable paper towels. The sterile insemination pipette was then guided manually through the cervix and advanced into the uterine body.

### Endometrial sampling and processing

Endometrial fragments were collected from the body–cornual junction using uterine biopsy forceps (Madoz et al., 2014). Biopsies were performed immediately before and 15 days after uterine infusion (D0 and D15, respectively). Each endometrial fragment was divided into two samples for analysis by light microscopy and scanning electron microscopy (SEM).

For light microscopy, samples were fixed in 10% neutral buffered formalin and processed for embedding in a glycol methacrylate‑based plastic resin. Sections were cut and stained with hematoxylin and eosin. For SEM, samples were fixed in 2.5% glutaraldehyde in 0.1 M sodium cacodylate buffer, washed in 0.1 M sodium cacodylate buffer, and dehydrated through a graded ethanol series to absolute ethanol. Finally, samples were coated with a thin layer of gold (20–30 nm thick) using an evaporation system (Sputter Coater SCD 050, BalTec).

### Light microscopy

Light microscopy was used to assess histomorphometric and histomorphological parameters of the endometrium. Microscopic analyses were performed by two specialist pathologists who were blinded to the treatments.

**Figure 1:**
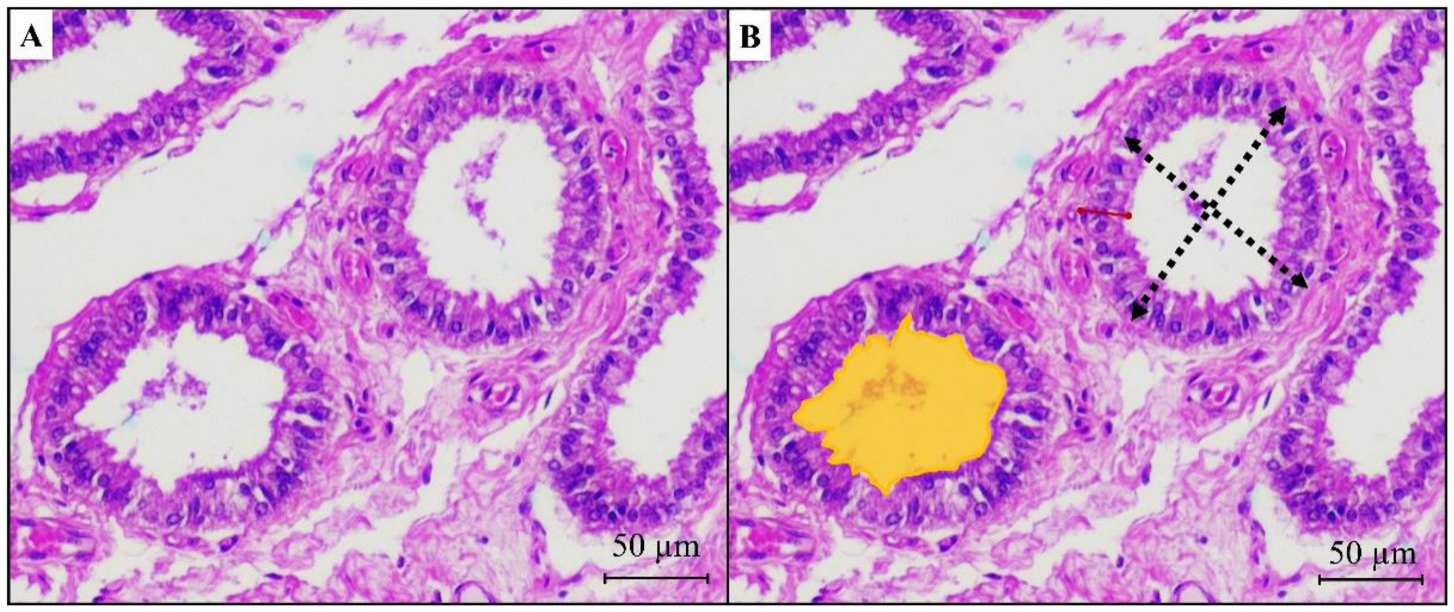
Histomorphometric analysis of the bovine endometrial stroma. Original and color‑marked light microscopic images (A and B, respectively) are shown. Measurements of glandular luminal area (yellow), height of the glandular epithelium (black line) and gland diameter (black dotted line) were measured immediately before and 15 days after treatment (H‑E, 100×).

Histomorphometric procedures were conducted as previously described by Ferreira et al. (2021a). Five fields from each endometrial sample were photographed using a digital image analysis system (Exfocus – 0.5×) connected to an Opticam 0400S optical microscope. Six histomorphometric parameters were measured using the Java‑based image processing program ImageJ (NIH): height of the endometrial epithelium, height of the glandular epithelium, glandular diameter, glandular luminal area, number of endometrial glands, and number of cells per endometrial gland (Figure 1). The tracing mode was used for measurements of glandular luminal area. Glandular epithelial height was determined by considering the basal and apical membranes of spherical spongy gland cells. The mean of the five largest endometrial glands per field was used to calculate glandular parameters.

Histomorphology was assessed using a scoring system adapted from Meira et al. (2012). Scoring was performed for the surface epithelium, lamina propria, endometrial glands, and vascular inflammatory status (Table 1). Six randomly selected regions of interest (ROIs) were examined from each sample, and the score assigned to each parameter corresponded to the mean of the six ROIs.

**Table 1:** Histopathological criteria for analysis of the endometrium from postpartum cows, per high‑power field at the stated magnification (ppf; ×10 or ×40). Adapted from Meira et al. (2012).

**Variable**	**Category**
Epithelium	
*i. Height*	columnar
	cuboidal
	flattened
*ii. Epithelial damage*	absent
	mild
	moderate
*iii. Inflamatory cell type*	absent
	mononuclear
	polymorphonuclear
*iv. Infiltrate intensity*	absent
	mild (≤5 cells/hpf; x40)
	moderate (≥6-10cells/hpf; x40)
	severe (>10 cells/hpf; x40)
Lamina propria	
*i. Inflamatory cell type*	absent
	mononuclear
	polymorphonuclear
*ii. Infiltrate intensity*	normal (≤20 cells/hpf; x40)
	mild (≤21-40 cells/hpf; x40)
	moderate (≥41-70cells/hpf; x40)
	severe (>70 cells/hpf; x40)
*iii. Lynphocytic aggregates*	absent
	mild (≤3 aggregates/hpf; x40)
	moderate (≥4-5 aggregates/hpf; x40)
	severe (>6 aggregates/hpf; x40)
Endometrial gland	
*i. Atrophy or dilatation*	absent
	present
*ii. Fibrosis*	mild (1-3 layers/hpf; x40)
	moderate (4-5 layers/hpf; x40)
	severe (>6 layers/hpf; x40)
Vascular	
*i. Vessel degeneration*	absent
	present
*ii. Hemorrhage*	absent
	present
*iii. Hemosiderin macrophages*	absent
	present

Scanning electron microscopy (SEM).

SEM was performed using a Quanta 200 system (FEI Company) operated with a 30 kV tungsten filament, secondary electron detector, and EDS microanalysis capabilities. Four magnifications were used for image acquisition (500×, 1500×, 1500×, and 3000×), and morphological characteristics were documented in TIFF format. The lowest magnification was used to select areas for subsequent measurements at intermediate magnifications, whereas detailed images were captured at the highest magnification. SEM images were evaluated for the presence of cilia and microvilli on the apical surface of the epithelium.

### Uterine cytology

Uterine cytology was performed immediately before each uterine biopsy (D0 and D15) using a gynecological cytobrush attached to sterile cytology forceps (Kasimanickam et al., 2004). Once the cervix was passed, the cytobrush was exposed, rolled along the endometrium, and then retracted into the protective forceps. After collection, the cytobrush was removed from the pistol grip and rolled onto a microscope slide. Slides were stained using a rapid Panótico stain (Laborclin, Brazil), according to the manufacturer’s instructions.

The percentage of polymorphonuclear neutrophils was determined by counting 200 cells per immersion field (1000× magnification). A cut‑off value of ≥8% PMN was used for diagnosing subclinical endometritis, as previously described for postpartum cows (Madoz et al., 2013).

### Antimicrobial analysis

Antibiotic sensitivity testing (AST), minimum inhibitory concentration (MIC), and minimum bactericidal concentration (MBC) assays were performed to assess the antimicrobial efficacy of ozonated sunflower oil against bacteria isolated from the uterine lumen of cows at D0. Four bacterial species were isolated: *Streptococcus* spp., *Staphylococcus* spp., *Escherichia coli* and *Arcanobacterium pyogenes*.

AST consisted of measuring the diameter of bacterial inhibition zones around antibiotic disks and comparing the results with interpretive criteria from the Clinical and Laboratory Standards Institute (CLSI, 2024). MIC determination followed CLSI guidelines (2024), with adaptations that used resazurin as an indicator of microbial activity (Sarker et al., 2007). MIC and MBC assays were carried out to distinguish bactericidal from bacteriostatic effects of the treatments in accordance with CLSI guidelines (2024). Similar MIC and MBC values indicated bactericidal activity of a given treatment against the bacteria tested, whereas divergent values suggested a bacteriostatic effect.

### Data analyses

Data were initially tested for normality using the D’Agostino–Pearson test. For histomorphometric variables and percentages of neutrophils, a two‑way repeated‑measures ANOVA was used and, when a significant interaction was detected, Bonferroni‑adjusted post hoc test was performed. Non‑normally distributed continuous data were analyzed using Friedman test for repeated measures within groups and Mann–Whitney U test for comparisons between groups. Histomorphological scores were compared between groups and time points using non‑parametric tests. Categorical variables were analyzed using chi‑square tests of independence. A probability of P≤0.01 was considered statistically significant. Data are presented as mean±S.E.M.

## Results

Regardless of treatment and time, all uterine samples exhibited a simple columnar epithelium supported by a network of collagen fibers, with mild to moderate epithelial detachment. The absence of active inflammatory cells or lymphocyte aggregates adjacent to the detached epithelium suggests that this detachment was artefactual. In both groups, a severe epithelial inflammatory infiltrate was observed at D0, whereas only a mild infiltrate was present at D15. At D0, cows from both groups showed similar intensities of granulomas and hemorrhagic spots in the lamina propria. However, only uterine samples from the O_3_ group showed a marked reduction in clusters of white blood cells and blood extravasation at D15, indicating a treatment‑by‑time interaction for these inflammatory features.

Marked hemorrhage and vascular dilation at D15 were noted only in cows from the non‑O_3_ group, whereas greater stromal accumulation between endometrial glands was observed 15 days after ozonated oil infusion, further supporting differential temporal responses between treatments. Histopathological changes associated with endometrial degeneration, such as periglandular fibrosis and endometrial atrophy, were not detected in the uteri of either group.

**Figure 2:**
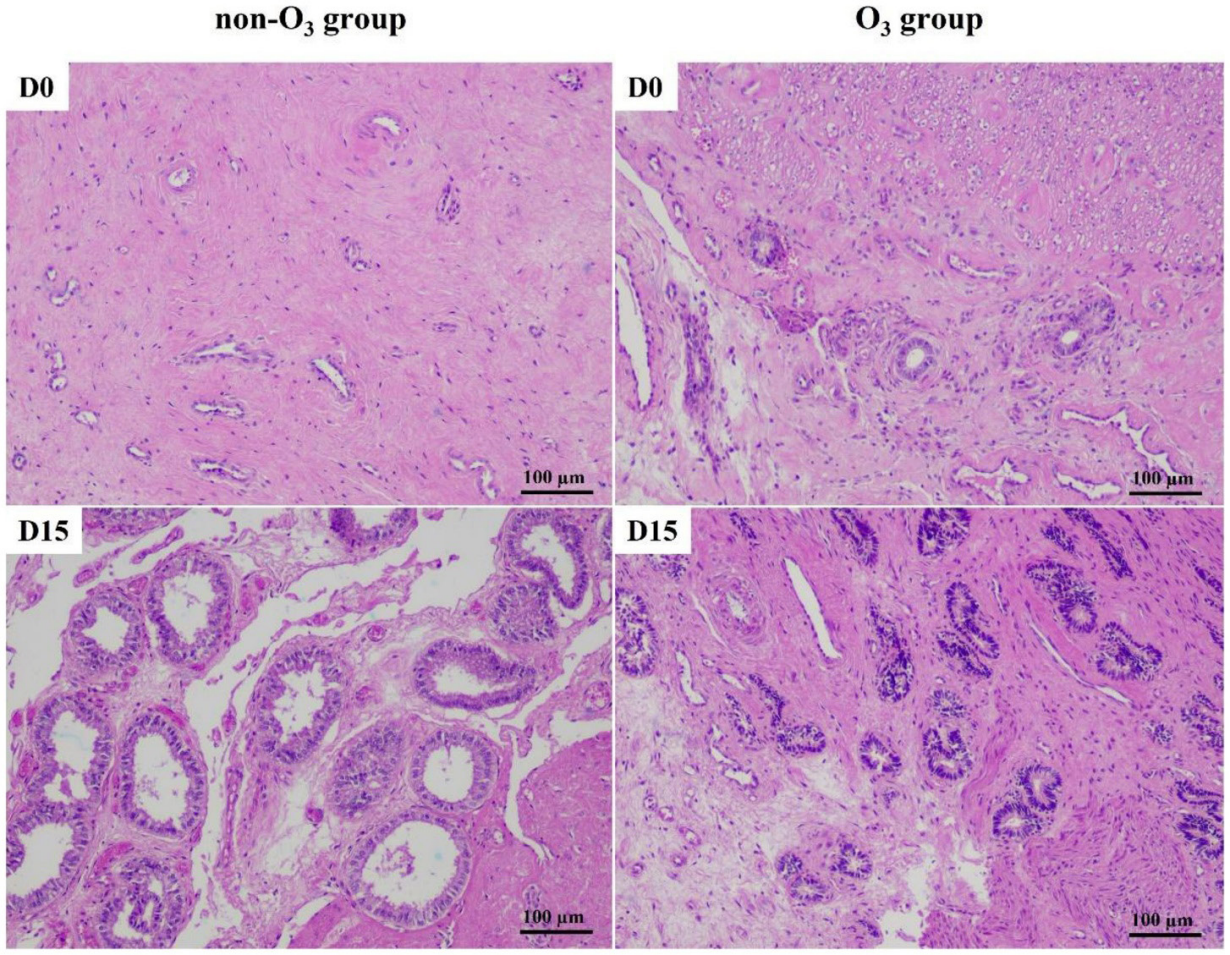
Histological sections of endometrial stroma from postpartum cows immediately before (D0) and 15 days (D15) after uterine infusion of non‑ozonated or ozonated oil (non‑O_3_ and O_3_ groups, respectively). Endometrial gland dilatation are visualized in non‑O_3_ group (H‑E, 100×).

For the number of endometrial glands, a significant main effect of time (P<0.10) and a significant treatment‑by‑time interaction (P<0.01) were detected. Before treatment (D0), both groups had few visible glands (5.6±0.6 glands; P>0.10), whereas at D15 a significant proliferation was observed in both groups. The increase in gland number at D15 was approximately three‑fold greater in uteri exposed to ozonated oil than in the non‑O_3_ group (32.6±2.5 vs. 11.0±1.0 glands, respectively; P<0.001; Table 2). Glandular epithelial height showed no significant main effects of treatment or time and no interaction (P>0.10). In contrast, histomorphometric analysis demonstrated a significant effect of treatment and a treatment‑by‑time interaction (both P<0.01) for gland dimensions (Table 2; Figure 2). After ozonated oil infusion, only a minor increase in glandular luminal area was detected between D0 and D15 in the O_3_ group (P<0.10), whereas the non‑O_3_ group exhibited an approximately eight‑fold expansion at D15 compared with D0 (P<0.01). Similarly, the number of cells per endometrial gland increased significantly over time only in the non‑O_3_ group (P<0.01), whereas no temporal change was detected in the O_3_ group (Table 2).

**Table 2:** Endometrial histomorphometry in puerperal primiparous cows receiving intrauterine infusion of ozonated or non‑ozonated sunflower oil (O_3_ and non‑O_3_ groups; n=9 cows/group). Sampling immediately before and 15 days after treatment corresponded to D0 and D15, respectively. Different superscript letters (a, b and c) within a parameter indicate significant differences (P<0.01).

**Parameter**	**O_3_ group**		**non-O_3_ group**	
	**D0**	**D15**	**D0**	**D15**
Number of glands	5.1±0.5^a^	32.6±2.5^c^	6.1±2.2^a^	11.0±1.0^b^
Number of cells per gland	33.2±1.3^a^	34.1±2.2^a^	26.7±1.2^a^	57.7±2.9^b^
Epithelial height (µm)	67.3±6.5	57.9±5.0	44.1±4.1	54.3±3.7
Gland height (µm)	33.2±1.3	34.1±2.2	33.2±2.0	30.9±2.7
Gland diameter (µm)	111.0±10.9^a^	111.9±10.8^a^	108.3±5.5^a^	2013.5±20.9^b^
Gland luminal area (µm^2^)	1750.6±303.2 ^a^	2376.6±830.7 ^b^	1713.2±300.9 ^a^	15008.9±1757.0 ^c^

**Figure 3:**
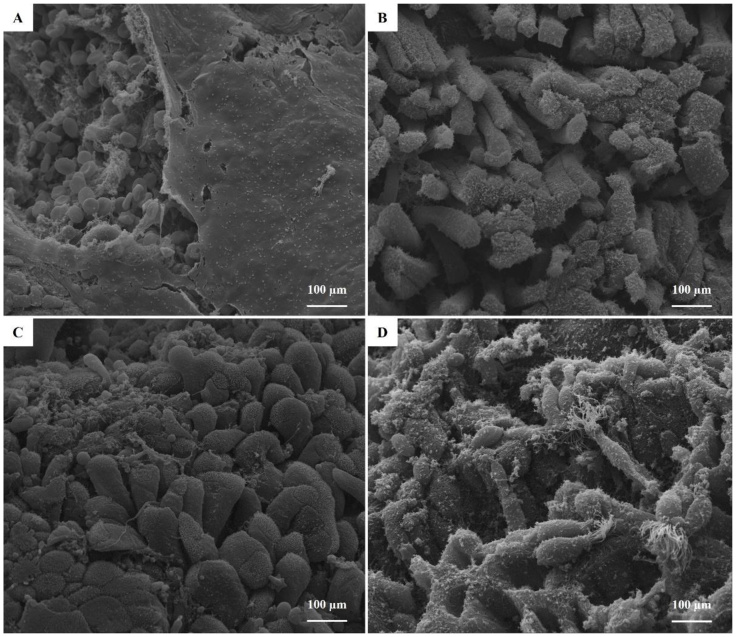
Scanning electron microscopy of the apical surface of the luminal uterine epithelium of the puerperal uterus immediately before (A and C) and 15 days after (B and D) a single infusion of ozonated oil (O_3_ group) in primiparous postpartum cows.

**Figure 4:**
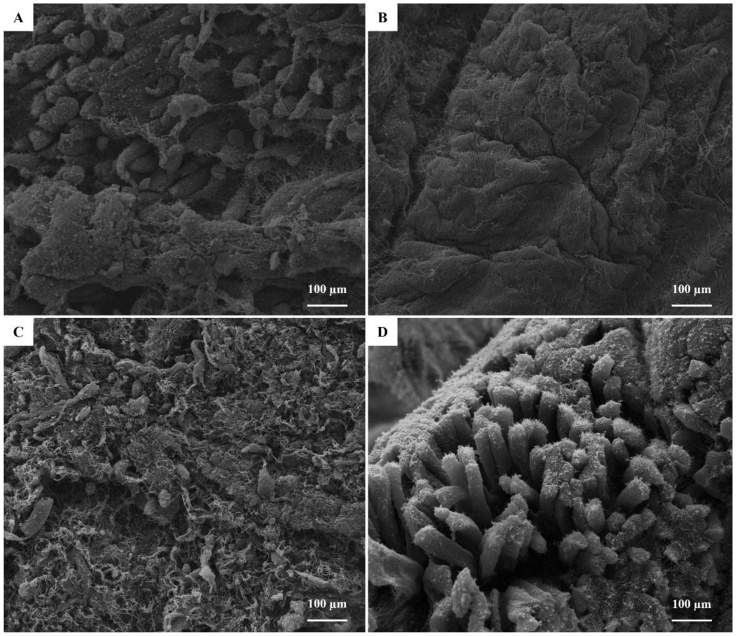
Scanning electron microscopy of the apical surface of the luminal uterine epithelium of the puerperal uterus immediately before (A and C) and 15 days after (B and D) a single infusion of non‑ozonated oil (non-O_3_ group) in primiparous postpartum cows.

SEM provided detailed images of the apical surface of epithelial cells in all samples. Qualitative SEM analysis revealed no morphological abnormalities attributable to uterine infusion of ozonated sunflower oil (Figures 3 and 4). Cells bearing microvilli and cilia were identified in both groups at both time points. Microvilli‑covered cells predominated; however, within the same tissue section, some areas showed nearly all cells with microvilli, whereas others displayed a cratered apical surface with microvilli lining the junctions between cells. Approximately 30% of fields showed <50% microvillar coverage. The presence of ciliated cells varied and was sporadic across evaluation times. No apical protrusions corresponding to pinopodes were detected.

For uterine cytology, analysis of PMN percentage indicated a significant main effect of time (P<0.01), but no effect of treatment and no treatment‑by‑time interaction (P>0.10). The non‑O_3_ and O_3_ groups had similarly high PMN percentages at D0 (47.3±17.9% and 45.1±17.0%, respectively; P>0.10). By D15, PMN percentages had decreased markedly in both groups (5.9±3.3%; P<0.01 vs. D0), with no difference between treatments at this time point (P>0.10). At D15, only one cow from each group exceeded the ≥8% cut‑off for subclinical endometritis (17.5% and 45% in the O_3_ and non‑O_3_ groups, respectively).

AST showed that ozonated sunflower oil was effective against both Gram‑positive and Gram‑negative bacteria (Table 3). Compared with tetracycline, ozonated sunflower oil produced larger inhibition zones for all bacterial strains tested. Furthermore, ozonated sunflower oil exhibited bactericidal activity at concentrations below 1.50 µg mL^‑1^ (Table 4), whereas non‑ozonated sunflower oil did not show antimicrobial activity. MIC and MBC values for *Streptococcus* spp., *Staphylococcus* spp., *E. coli* and *A. pyogenes* are presented in Table 4.

**Table 3:** Antibiotic sensitivity testing (AST) based on the diameter of inhibition zones of ozonated and non‑ozonated sunflower oils (O_3_ and non‑O_3_ oils, respectively) against bovine uterine bacteria. Tetracycline and untreated samples were considered control groups. Susceptibility and resistance of the bacteria to the treatments are indicated by the letters “S” and “R”, respectively.

**Bacteria**	**Diameter of zone inhibition (mm)**			
	**O_3_ oil**	**non-O_3_ oil**	**Untreated**	**Tetracycline**
*Escherichia coli*	33.5±3.5 (S)	0.0±0.0 (R)	0.0±0.0 (R)	16.7±1.8 (S)
*Staphylococcus* ssp.	41.3±4.9 (S)	0.0±0.0 (R)	0.0±0.0 (R)	24.3±2.3 (S)
*Streptococcus* ssp.	38.0±1.2 (S)	0.0±0.0 (R)	0.0±0.0 (R)	14.3±2.6 (S)
*Arcanobacterium pyogenes*	33.2±1.3 (S)	0.0±0.0 (R)	0.0±0.0 (R)	23.7±2.2 (S)

**Table 4:** Minimum inhibitory and bactericidal concentrations (MIC and MBC; µg mL^‑1^) of ozonated and non‑ozonated sunflower oils (O_3_ and non‑O_3_, respectively) against bovine uterine bacteria (*Escherichia coli*, *Staphylococcus* spp., *Streptococcus* spp. and *Arcanobacterium pyogenes*).

**Bacteria**	**O_3_ oil**		**non-O_3_ oil**		**untreated**		**Tetracycline**	
	**MIC**	**MBC**	**MIC**	**MBC**	**MIC**	**MBC**	**MIC**	**MBC**
*E. coli*	0.05	0.09	>5.9	>5.9	>5.9	>5.9	0.05	0.09
*Staphylococcus* ssp.	0.09	0.18	>5.9	>5.9	>5.9	>5.9	0.18	0.36
*Streptococcus* ssp.	0.09	0.18	>5.9	>5.9	>5.9	>5.9	0.33	0.97
*A. pyogenes*	0.18	0.18	>5.9	>5.9	>5.9	>5.9	0.18	0.36

## Discussion

This present study demonstrates that a single intrauterine infusion of ozonated sunflower oil during early puerperium enhances uterine involution in primiparous cows by stimulating endometrial gland remodeling while preserving luminal ultrastructure and without inducing degenerative lesions. In addition, ozonated oil showed broad-spectrum bactericidal activity against major uterine pathogens, directly supporting the hypothesis that this therapy combines structural endometrial benefits with effective local antimicrobial action. Collectively, the in vivo and in vitro results suggest that this non-antibiotic approach may support uterine health and reproductive performance in the critical early postpartum period.

From a histomorphometric perspective, cows treated with ozonated oil exhibited a pronounced increase in the number of endometrial glands, together with smaller glandular diameter, reduced luminal area and fewer cells per gland, indicating intense glandular proliferation without cystic dilatation. This pattern contrasts with the structural changes typically associated with postpartum endometritis, in which glandular dilatation, epithelial necrobiosis, stromal edema and exudation are frequent (Suleymanov et al., 2018; Aires et al., 2025). The absence of periglandular fibrosis or endometrial atrophy suggests that the morphologic remodeling promoted by ozonated oil does not progress toward chronic degenerative lesions, which is an important safety consideration for any repeated intrauterine therapy (Kübar and Jalakas, 2002).

​Histopathological scoring further revealed a time-by-treatment interaction, in which only uteri exposed to ozonated oil showed a marked reduction in stromal leukocyte clusters and hemorrhagic foci at D15, whereas cows treated with non-ozonated oil still exhibited pronounced hemorrhage and vascular dilation. Persistent vascular congestion, hemorrhage and inflammatory infiltrate beyond the third postpartum week are strongly associated with delayed involution, higher bacterial load and impaired reproductive performance (Bondurant, 1999; Sheldon et al., 2009), highlighting the importance of rapid resolution of these lesions for uterine health. The attenuation of hemorrhage and inflammatory lesions in the lamina propria and vasculature of ozonated-oil–treated cows is consistent with the reported antioxidative, immunomodulatory and pro-angiogenic effects of O_3_ therapy, which include stimulation of fibroblasts and collagen synthesis (Xiao et al., 2021), reduction of genotoxic damage (Akdeniz et al., 2018), inhibition of NF-κB–mediated inflammation (Zeng et al., 2020) and improvement of local microcirculation (Ferreira et al., 2021a).

​ The apical surface may have a key role in governing pregnancy establishment (Kumro et al., 2020) SEM showed preserved apical morphology of luminal epithelial cells in O_3_ and non-O_3_ groups, with predominance of microvillus-bearing cells and sporadic ciliated cells, and no evidence of necrosis, massive desquamation or surface disruption attributable to ozonated oil. This preservation of luminal ultrastructure contrasts with the severe dystrophic and necrobiotic changes, destruction of microvilli, proliferation of coccoid microflora and nuclear edema described in cows with acute postpartum endometritis, conditions that compromise uterine receptivity and subsequent embryo survival (Suleymanov et al., 2018). Given that most conventional intrauterine therapies were not originally designed with preservation of endometrial ultrastructure in mind, the absence of detectable adverse structural effects after ozonated oil infusion strengthens its potential as a safe local treatment that does not interfere with epithelial remodeling required for future implantation.

​Independently of the treatment, the rapid mobilization of neutrophils to the postpartum uterus at D0 is considered a beneficial response for uterine well-being after calving (Santos et al., 2009; Gilbert and Santos, 2016). Furthermore, the marked decrease to values below the 8% threshold for subclinical endometritis at D15, without differences between both groups, is also consistent with the physiological trajectory of postpartum uterine inflammation in healthy cows (Foley et al., 2015; Sicsic et al., 2018). The discrepancy between similar cytological resolution and more favorable histological inflammatory scores in O_3_ group support low agreement between endometrial biopsy and cytology for subclinical endometritis diagnosis reported by Madoz et al. (2014).

​Taken together, the light microscopy, SEM and cytology findings suggest that intrauterine ozonated sunflower oil modulates the postpartum involution process by supporting orderly glandular proliferation, reducing hemorrhage and inflammatory lesions, and preserving luminal ultrastructure, without delaying neutrophil clearance. These observations are in line with experimental and clinical data in cows showing that ozonated derivatives can improve reproductive performance, shorten uterine regression and reduce the need for systemic antibiotics in cases of uterine diseases (Đuričić et al., 2012a, 2012b, 2014; Zobel et al., 2014; Escandón et al., 2020).

The in vitro antimicrobial assays demonstrated that ozonated sunflower oil exerted potent bactericidal effects against all postpartum uterine isolates tested with inhibition zones exceeding those of tetracycline and minimum inhibitory and bactericidal concentrations below 1.5 µg/mL, whereas non-ozonated oil showed no activity. These results corroborate previous evidence that ozonated vegetable oils possess broad-spectrum antimicrobial properties against bacteria (Ugazio et al., 2020), including multidrug resistant strains (Grandi et al., 2022), as well as biofilm (Higa et al., 2022), fungi (Celenza et al., 2020) and oomycetes (Ferreira et al., 2021b) relevant to veterinary practice. In the specific context of postpartum uterine disease, this broad spectrum is particularly relevant because *E. coli* and *A. pyogenes*, among others, act synergistically to establish metritis and endometritis and are frequently associated with virulence factors that aggravate tissue damage and reduce reproductive performance (Bicalho et al., 2016; Yamamura et al., 2022).

The germicidal action of ozonated oil is attributed to the formation of peroxides and ozonides during ozonation of unsaturated fatty acids (Hassan et al., 2021), which react with microbial cell envelopes (Duah Boakye et al., 2019), leading to non-specific oxidation of membrane lipids, glycoproteins and amino acids, increased permeability, enzyme inactivation and DNA fragmentation (Colombo et al., 2018). Unlike conventional antibiotics that target defined metabolic pathways or structural components and therefore exert strong selective pressure for resistance development (Uddin et al., 2021), the oxidative damage induced by ozonated oil is diffuse and multi-target, making it unlikely that bacteria will acquire stable, heritable mechanisms to evade its action (Vieira et al., 2025). This feature is especially valuable in dairy systems, where routine use of intrauterine or systemic antibiotics for endometritis has been linked to rising antimicrobial resistance (Murray et al., 2022) and concerns about drug residues in milk (Sachi et al., 2019), prompting calls for non-antibiotic alternatives in postpartum uterine therapy.

The postpartum uterus is almost universally contaminated by bacteria within the first two weeks after calving (Sheldon et al., 2009), yet only a subset of cows develop clinical or subclinical uterine disease, a difference that reflects complex interactions among pathogen virulence, bacterial load and host immune competence (Sheldon et al., 2019). Pathogenic strains of *E. coli*, *F. necrophorum* and *T. pyogenes* expressing virulence genes such as fimH, lktA and fimA/plo are strongly associated with metritis and endometritis and with impaired fertility (Bicalho et al., 2012), underscoring the need for therapeutic approaches capable of rapidly reducing or eliminating such organisms from the uterine lumen. The present findings show that ozonated sunflower oil meets this requirement in vitro for uterine pathogens, supporting its use either as a preventive intrauterine treatment in high-risk cows or as an adjunct to systemic therapy in clinically affected animals, while avoiding the drawbacks of conventional intrauterine antibiotic infusions.

Beyond direct bactericidal effects, ozone-based therapies have been reported to increase leukocyte phagocytic activity (Kucuksezer et al., 2014), modulate cytokine secretion (Delgado-Roche et al., 2017) and upregulate endogenous antioxidant defenses (Galiè et al., 2018), thereby enhancing the capacity of the innate immune system to clear residual pathogens and resolve inflammation. Clinical studies in cattle and small ruminants indicate that intrauterine O_3_ (gas or foam) can improve conception rates, reduce days open and enhance the cure rate of endometritis and retained placenta when compared with or combined with antibiotic protocols, without reported adverse local effects or residues in cattle (Escandón et al., 2020). In this context, ozonated vegetable oils emerge as a pragmatic, easy-to-handle and stable formulation (Dominguez Lacueva et al., 2025) that delivers the antimicrobial and immunomodulatory benefits of O_3_ directly to the uterine lumen, aligning with current priorities in animal reproduction to reduce antibiotic use and mitigate antimicrobial resistance while safeguarding reproductive efficiency.

## Conclusion

Infusion of ozonated sunflower oil during puerperium enhanced uterine involution in primiparous cows by increasing endometrial gland proliferation while preventing excessive glandular dilatation. Endometrial glands exposed to ozonated oil showed reduced diameter, luminal area and cell number per gland, and scanning electron microscopy confirmed preservation of the luminal ultrastructure after treatment. In parallel, ozonated oil exhibited broad‑spectrum bactericidal activity against major postpartum uterine pathogens, supporting its potential use as a non‑antibiotic intrauterine therapy for postpartum uterine disease in cattle.

## Data Availability

Research data is not available
